# The Peopling of Korea Revealed by Analyses of Mitochondrial DNA and Y-Chromosomal Markers

**DOI:** 10.1371/journal.pone.0004210

**Published:** 2009-01-16

**Authors:** Han-Jun Jin, Chris Tyler-Smith, Wook Kim

**Affiliations:** 1 Department of Biological Sciences, Dankook University, Cheonan, Korea; 2 The Wellcome Trust Sanger Institute, Wellcome Trust Genome Campus, Hinxton, Cambridge, United Kingdom; Louisiana State University, United States of America

## Abstract

**Background:**

The Koreans are generally considered a northeast Asian group because of their geographical location. However, recent findings from Y chromosome studies showed that the Korean population contains lineages from both southern and northern parts of East Asia. To understand the genetic history and relationships of Korea more fully, additional data and analyses are necessary.

**Methodology and Results:**

We analyzed mitochondrial DNA (mtDNA) sequence variation in the hypervariable segments I and II (HVS-I and HVS-II) and haplogroup-specific mutations in coding regions in 445 individuals from seven east Asian populations (Korean, Korean-Chinese, Mongolian, Manchurian, Han (Beijing), Vietnamese and Thais). In addition, published mtDNA haplogroup data (N = 3307), mtDNA HVS-I sequences (N = 2313), Y chromosome haplogroup data (N = 1697) and Y chromosome STR data (N = 2713) were analyzed to elucidate the genetic structure of East Asian populations. All the mtDNA profiles studied here were classified into subsets of haplogroups common in East Asia, with just two exceptions. In general, the Korean mtDNA profiles revealed similarities to other northeastern Asian populations through analysis of individual haplogroup distributions, genetic distances between populations or an analysis of molecular variance, although a minor southern contribution was also suggested. Reanalysis of Y-chromosomal data confirmed both the overall similarity to other northeastern populations, and also a larger paternal contribution from southeastern populations.

**Conclusion:**

The present work provides evidence that peopling of Korea can be seen as a complex process, interpreted as an early northern Asian settlement with at least one subsequent male-biased southern-to-northern migration, possibly associated with the spread of rice agriculture.

## Introduction

An understanding of the evolutionary history of East Asian populations has long been a subject of interest in the field of human evolutionary genetics. Based on results of classical genetic markers, there is significant separation between southern and northern populations of East Asia [1]. This north-south genetic differentiation is likely to have an origin in the early peopling of the region. There have been two major models for early migration routes into East Asia. The first model postulates a southeast Asian origin, followed by a northward migration [2]. Recent genetic surveys using autosomal microsatellite markers [3] and Y-chromosomal binary markers [4] have been interpreted as supporting this model. In contrast, the second model suggests a multidirectional route: one migration through central Asia and one through southeast Asia [1], [5], [6]. Thus, understanding the genetic origin and history of Korea may be informative for questions concerning prehistoric migration route(s) and population expansions in East Asia.

The Korean Peninsula is located to the north of the Yellow and Yangtze Rivers of China, and bounded to the northeast by Russia. Therefore, the Koreans are geographically a northeast Asian group. Anthropological and archeological evidence suggests that the early Korean population was related to Mongolian ethnic groups who inhabited the general area of the Altai Mountains and Lake Baikal regions of southeast Siberia [7]. According to Korea's founding myths, the Ancient Chosun (the first state-level society of Korea) was established around 2,333 BC in the region of southern Manchuria but later moved into the Pyongyang area of northwest Korea. In addition, archeological evidence reveals that rice cultivation had spread to most parts of the Korean Peninsula by around 1,000–2,000 BC, introduced from the Yellow River and/or Yangtze River basin in China [8].

Studies of classical genetic markers showed that Koreans tend to have a close genetic affinity with Mongolians among East Asians [9]–[11]. In contrast, recent surveys of Y-chromosomal DNA variation revealed that the Korean population contained lineages typical of both southern and northern East Asian populations [6], [12], [13]. The Koreans appeared to have affinities with Manchurians, Yunnan-Chinese from southern China, and Vietnamese [13].

To understand the genetic history of Korea better, more data from additional genetic markers from Korea and its surrounding regions are necessary. Mitochondrial DNA (mtDNA), like the Y chromosome, can also provide valuable information about the phylogeography of human populations due to its special features of haploidy and uniparental inheritance [14]–[18]. Although recent investigations of mtDNA variation in East Asia have provided valuable information for constructing a robust phylogenetic tree of mtDNA haplotypes, limited data on the Korean population are available [19]–[21].

In this study, we present new data on the mtDNA sequence variation of the hypervariable segments I and II (HVS-I and HVS-II) and haplogroup-specific mutations in coding regions in 445 individuals from seven East Asian populations, including Korea. In addition, mtDNA haplogroup data (N = 3307), mtDNA HVS-I sequences (N = 2313), Y chromosome haplogroup data (N = 1697) and Y chromosome STR data (N = 2713) from the literature were analyzed to elucidate wider aspects of the genetic structure of East Asian populations.

## Materials and Methods

### DNA samples and reference data

We analyzed a total of 445 individuals, collected from seven East Asian populations (Korean, Korean-Chinese (People of Korean origin now living in China), Mongolian, Manchurian, Chinese Han (Beijing), Vietnamese, and Thai). The DNA samples included subsets of the samples examined by Jin et al. [13] and Kwak et al. [22], although the exact number of subjects for each population occasionally varies between these studies. In addition, we included the following new Korean-Chinese and Mongolian samples: 51 Korean-Chinese from northern China and 47 Mongolians from Ulaanbaatar. This study was approved by the Ethics Committee and institutional review boards of Institute of Bio-Science and Technology in the Dankook University in Cheonan, and separate written informed consent was obtained for enrollment from all participants. DNA was prepared from whole blood by the standard method [23] or was extracted from buccal cells according to the procedure of Richards et al. [24].

**Figure 1:**
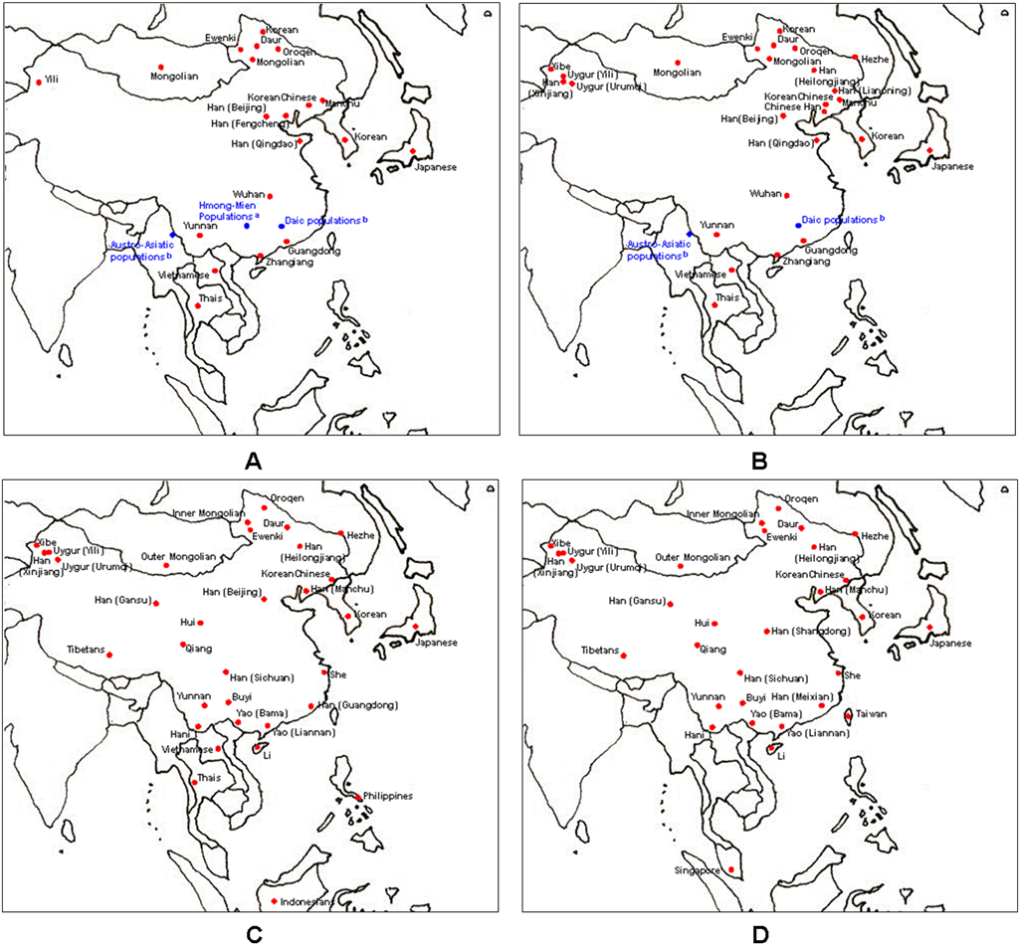
Geographic locations of the Asian populations studied. (A) mtDNA haplogroups. (B) mtDNA HVS-I sequences. (C) Y-chromosome haplogroups. (D) Y-chromosome STRs. ^a^Wen et al. [27]. ^b^Li et al. [36].

In addition to our mtDNA data sets, mtDNA haplogroup data for 2862 individuals, mtDNA HVS-I sequences data for 1868 individuals, Y chromosome haplogroup data for 1697 individuals and Y chromosome STR data (ten Y-STR loci: DYS19, DYS389I, DYS389b, DYS390, DYS391, DYS392, DYS393, DYS437, DYS438 and DYS439) for 2716 individuals were retrieved from the literature [19], [20], [25]–[40] to elucidate the genetic relationship between Koreans and other East Asian populations (details in Supplementary Table S1 and Figure 1). For mtDNA haplogroup analysis, some sub-haplogroups were clustered into major haplogroups according to their phylogenetic affiliations (Supplementary Table S2). Similarly, Y chromosome haplogroups from reference data were reclassified into a common set of 13 Y-chromosomal (sub)haplogroups that captured most of the phylogenetic information to allow population comparisons (Supplementary Table S3).

### PCR amplification

PCR amplification of the HVS-I and HVS-II of mtDNA control region was performed using two primer sets as described by Yao et al. [19]: HVS-I, L15996/H16498 (nucleotide positions, 15975-15996/16517-16498); HVS-II, L29/H408 (nucleotide positions, 8-29/429-408). Primers were designed for amplifying multiple fragments that contain haplogroup diagnostic polymorphisms in the coding regions [19], [20], [41]. Each set of segments was amplified in a 50 ul reaction containing 25 ng of genomic DNA, 10 pM of each primer, 0.2 mM of dNTPs, 2.0 mM MgCl_2_, 5 mM KCl, 10 mM TRIS-HCl (pH 8.3) and 1.5 U AmpliTaq DNA polymerase (Perkin-Elmer, CA, USA). The PCR amplification was carried out using a GeneAmp® PCR system 9700 thermal cycler (Applied Biosystems, CA, USA) under the conditions described in Table 1.

**Table 1:** Primers for mtDNA amplification, sequencing and RFLP analyses.

Primer Pair	Primer sequences (5′ to 3′)	Annealing Temperature (°C)	Polymorphisms at/in
L29/H408	GGT CTA TCA CCC TAT TAA CCA C	54	HVS-II
	CTG TTA AAA GTG CAT ACC GCC A		
L4499/H5099	TGG CCC AAC CCG TCA TCT AC	60	+4831 *Hha*I(4833)
	GGA ATG CGG TAG TAG TTA GG		
L4887/H5442	TGA CAA AAA CTA GCC CCC ATC T	60	5176 *Alu*I (5178A)
	GCG ATG AGT GTG GGG AGG AA		
L8215/H8297	ACA GTT TCA TGC CCA TCG TC	57	9-bp deletion
	ATG CTA AGT TAG CTT TAC AG		
L10170/H10660	ACA TAG AAA AAT CCA CCC CTT ACG	59	10171-10659
	TTC GCA GGC GGC AAA GAC TA		
L12114/H12338	CTC AAC CCC GAC ATC ATT ACC	58	12308
	ATT ACT TTT ATT TGG AGT TGC ACC AAA ATT		
L14054/H14591	TCA CAG CAC CAA ATC TCC AC	57	14178, 14308, 14318, 14470
	AAG CCT TCT CCT ATT TAT GG		
L14575/H15086	ACC CGA CCA CAC CGC TAA CA	57	14668, 14766, 14783, 15043
	AGG AGG ATA ATG CCG ATG TT		
L15996/H16498	CTC CAC CAT TAG CAC CCA AAG C	60	HVS-I
	CCT GAA GTA GGA ACC AGA TG		

PCR conditions were 94°C for 5 min, for denaturation; 94°C for 45 sec; annealing temperature shown for 45 sec, for amplification; and 72°C for 1 min, for 35 cycles; incubation at 72°C for 5 min.

### mtDNA sequencing and genotyping of RFLP

After PCR amplification, each PCR product was purified using the Wizard® PCR Preps DNA Purification System (Promega, WI, USA) and then sequenced by cycle sequencing using either a MegaBase 1000 sequencer (Amersham Bioscience, USA) or an ABI PRISM™ 310 Genetic Analyzer (Applied Biosystems, CA, USA) with DYEnamic ET Dye Terminator (Amersham Bioscience, USA) or BigDye™ Terminator (PE Biosystems, USA), respectively. DNA sequences of the PCR amplicons were determined from both forward and reverse sequence data using the original primer pairs. The sequences from nucleotide position (np) 16024 to 16365 in HVS-I and from 73 to 340 in HVS-II were determined, since ambiguous electropherograms for 20–30 nucleotides near the primers were frequently observed.

The intergenic COII/tRNA^Lys^ 9-bp deletion was analyzed as described in Jin et al. [42]. In addition, several amplified segments, mainly in the mtDNA coding regions, were analyzed by RFLP typing and additional sequencing, as listed in Table 1.

### Sequence alignment and haplogroup analyses

Sequences were aligned and compared with the revised Cambridge Reference Sequence (rCRS) [43] using the Sequencher program ver. 2000 (Gene Codes corporation, MI, USA). The results were converted into a Microsoft Excel table (Microsoft Corporation, CA, USA). The mtDNAs were classified into the (sub-)haplogroups based on HVS-I/II motifs of haplogroup specific-sequences as well as coding regions as described in recent surveys [19], [20], [25], [44], [45]. The HVS-I motif searching and haplogroup-directed comparison with closely related sequences from other databases led us to tentatively assign each mtDNA to a haplogroup. To further characterize the mtDNA lineage tested, we compared their HVS-II motif to verify the predicted haplogroup status of each mtDNA. In general, more than 95% of mtDNA lineages can faithfully be classified to specific haplogroups using HVS-I/II motifs without extra information from coding region sequences [44]. However, in the remaining cases, their (sub-)haplogroups were characterized using sequence information from some coding region sites (Table 1). After each mtDNA was assigned to the most-derived named haplogroup, the haplogroup distribution frequencies in each of seven populations were estimated. For quality assurance purposes, we performed quasi-median network analysis [46], [47]. The HVS-I (np 16024–16365 np) and HVS-II (np 74–340) sequence of 445 individuals of this study have been submitted to GenBank (Accession Numbers, FJ493775-FJ494664).

### Data analyses

The genetic differentiation between different population samples and its statistical significance were assessed via *F*_ST_ (mtDNA HG and HVS-I/II and Y-SNPs) and *R*_ST_ (Y-STRs) values. The population genetic structure of the ethnic and/or regional groups was analyzed through the analysis of molecular variance (AMOVA) approach [48]. The calculations of diversity indices, *F*_ST_, *R*_ST_ and AMOVA were performed using the Arlequin 2.000 package [49]. Population pairwise *F*_ST_ and *R*_ST_ values were visualized by multidimensional scaling (MDS) plot analyses using SPSS 12.0 software.

Haplogroup-specific median-joining networks [50] for Y chromosome data were constructed using the NETWORK 4.2 program (www.fluxus-technology.com). Such networks were initially highly reticulated, and we reduced reticulations by first weighting the loci according to the inverse of their variance in the dataset used [51] and subsequently constructing a reduced-median network [52] to form the input of the median-joining network [53].

The admixture proportions of northeast Asian and the southeast Asian parental populations in the Korean population were estimated for mtDNA and the Y chromosome using the Admix 2.0 software [54]

## Results and Discussion

Almost all of the mtDNA lineages analyzed here could be assigned to the East Asian-specific (sub)haplogroups described recently [19], [20], [25], [44], [45], with the exception of two individuals belonging to the European mtDNA haplogroups T (Manchurian) and U5a (Mongolian) (Table 2). The gene diversity (*H*), nucleotide diversity (π_n_), and mean number of pairwise differences of the population samples are listed in Table 3. All seven populations displayed high levels of genetic diversity (*H*>0.99), suggesting a relatively large population size and heterogeneity of each mtDNA pool. The haplogroup frequencies observed in each population are summarized in Table 2. Based on these haplogroup assignments, the Koreans share lineages with both the southern and the northern haplogroup complexes of East Asia. We first attempted to quantitate these contributions by a detailed consideration of the distribution of each lineage.

**Table 2:** Distribution of mtDNA haplogroup frequencies in 7 East Asian populations.

Haplogroup	Korean-Chinese	Mongolian	Manchurian	Han (Beijing)	Vietnamese	Thais	Korean
A			3	1			3
A4	4	2	1	1	1		6
A5	1				1		5
A5a							1
B				2		1	
B4		2	2		3		7
B4a	2	1	1		1		11
B4b	2			2	1		
B4b1							4
B4c							1
B5a	1				1	3	2
B5b	1	1					2
C	1	8	1		2	4	3
C3		2					
D	1					2	1
D4	11	5	8	5	7	1	44
D4a	3			2			3
D4b		1					3
D5	2		1	3	1		6
D5a			1	2	1		3
F	1		3	3	1		
F1a	1	3	2	4	10		8
F1b	1	3	2	2		8	8
F1c	1		1				
F2							2
F2a		1					
G		2		1			1
G1a			3		1		1
G2	1			1			7
G2a	1	5	1	2	6		
G3		1			1		4
M	3	1			1	5	1
M7a1							7
M7b		1		1		3	
M7b1			2			2	1
M7b2	2					1	4
M7c		1	1		1		1
M7c1	1		1	1	1	1	6
M8a		1	1		1	1	2
M9a	1		1		1		3
M10	2				1	3	1
M11	1						1
N							1
N9a	5	2	2	3	3	1	12
R						2	
R11	1						
T			1				
U5a		1					
Y1		1					1
Y2							1
pre-Z		1					
Z		1	1	4	1	2	1
n	51	47	40	40	42	40	185
Total	445						

**Table 3:** Diversity indices of mtDNA in seven east Asian populations.

	Haplogroup data	Sequence data (HVS-I/II^a^)		
	Gene diversity	Gene diversity	Pairwise difference	Nucleotide diversity
Korean	0.9239+/−0.0132	0.9988+/−0.0007	10.07+/−4.62	0.039+/−0.020
Korean-Chinese	0.9357+/−0.0219	0.9992+/−0.0041	10.21+/−4.74	0.039+/−0.020
Mongolian	0.9454+/−0.0172	0.9991+/−0.0046	10.80+/−5.00	0.042+/−0.021
Manchurian	0.9462+/−0.0221	0.9974+/−0.0063	10.88+/−5.05	0.042+/−0.022
Han (Beijing)	0.9526+/−0.0135	1.0000+/−0.0056	11.38+/−5.27	0.044+/−0.022
Vietnamese	0.9152+/−0.0290	0.9919+/−0.0079	9.66+/−4.52	0.037+/−0.020
Thai	0.9269+/−0.0214	1.0000+/−0.0056	11.53+/−5.33	0.045+/−0.023

a HVS-I: np 16024–16365; HVS-II: np 73–340.

The highest (23.8%) frequency in the Korean mtDNA pool was observed for haplogroup D4, which is widespread in northern East Asia and especially in the Korean-Chinese (21.6%), and Manchurians (20.0%). In total, haplogroup D lineages including the subhaplogroups (D4, D4a, D4b, D5, and D5a) accounted for 32.4% of the Korean mtDNA pool. In addition, the Koreans present moderate frequencies of (sub)haplogroup A (8.1%) and (sub)haplogroup G (10.3%) lineages, mostly prevalent in northeast Asia and southeast Siberia [20], [55]–[57]. Other Siberian and Mongolian-prevalent haplogroups from the C, Y and Z lineages make up less than 4% of the Korean mtDNA pool. Haplogroups A5a and Y2 are found almost exclusively in Korea but were present at extremely low frequencies. In total, these northern haplogroups account for ∼60% of the mtDNA gene pool of the Koreans. In addition, southeast Asian-prevalent mtDNA lineages of (sub)haplogroups B (14.6%), M7 (10.3%), and F (9.7) are also found at moderate frequencies in the Korean population (Table 2). These findings suggest that more than 30% of the Korean mtDNA pool is attributable to maternal lineages with a more southern origin. We also found the haplogroup M7a1 exclusively in the Korean population. This result is consistent with previous reports that haplogroup M7a is restricted to Japan and south Korea [18], [20]. Thus, the distribution pattern of mtDNA haplogroups leads us to consider that the peopling of Korea is likely to have involved multiple sources.

**Figure 2:**
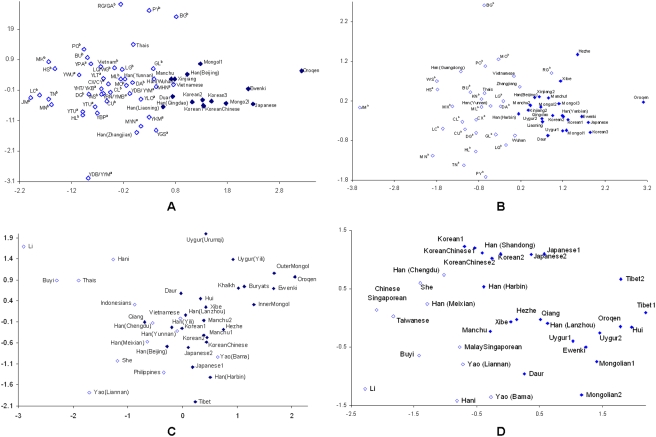
Multidimensional scaling (MDS) plot based on (A) *F*_ST_ distances of mtDNA haplogroups (stress = 0.21). (B) *F*_ST_ distances of mtDNA HVS-I sequences (stress = 0.19). (C) *F*_ST_ distances of Y chromosome haplogroups (stress = 0.21). (D) *R*_ST_ distances of Y chromosome STRs (stress = 0.19). (*closed diamond*: North Asians; *opened diamonds*: South Asians). ^a^Wen et al. [27]. ^b^Li et al. [36].

We then investigated the mtDNA and Y-chromosomal relationships between the East Asian populations, using both the new and published data. In these analyses mtDNA haplogroups, mtDNA HVS-I sequences, Y-SNPs and Y-STRs were compared (Supplementary Tables S1, S2, S3). Pairwise *F*_ST_ (mtDNA haplogroup, mtDNA HVS-I sequences and Y-SNPs) and *R*_ST_ (Y-STRs) values between East Asian populations were calculated (Supplementary Table S1). The *F*_ST_ distances of mtDNA markers (mtDNA haplogroups and HVR-I sequences) of Korean populations showed close relationships with Manchurians, Japanese, Mongolians and northern Han Chinese but not with southern Asians (Supplementary Tables S4 and S5; Figure 2A, B). In the MDS plots, the Korean samples lay entirely within the cluster of northern populations.

**Figure 3:**
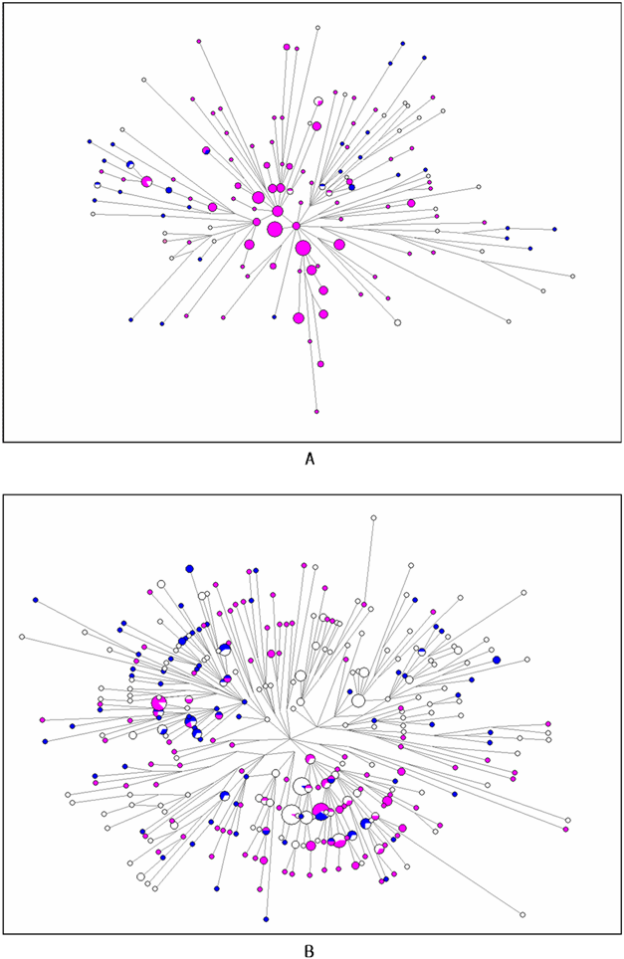
Median-joining network for east Asian (A) Network of 7 Y-STRs (DYS19, DYS389b, DYS389I, DYS390, DYS391, DYS392 and DYS393) variation within Haplogroup C. (B) Network of 7 Y-STRs within Haplogroup O3. Circle areas are proportional to haplotype frequency. Lines represent the mutational differences between haplotypes. The network corresponds the following colors: purple- far north Asian populations (Daur, Ewenki, Han (Xinjiang), inner Mongolians, Oroqen, outer Mongolians, Uygur (Yili), Uygur (Urumqi) and Xibe); blue- Koreans; white-far south Asian populations (Buyi, Han (Guangdong), Han (Sichuan), Han (Yunnan), Hani, Indonesians, Li, philippines, She, Thais, Vietnamese, Yao (Bama) and Yao (Liannan).

In contrast, the results of Y chromosome analyses (based on Y-SNPs and Y-STRs) of Korean populations revealed closer relationships with both northeast and southeast Asian populations (Supplementary Tables S6 and S7; Figure 2C, D). Like the mtDNA distances, Y-chromosomal distances from Manchurian, Japanese and northern Han Chinese populations were usually not significantly greater than zero, but some distances from southern Han populations (e.g. Yunnan Han, Y haplogroups; Meixian Han, Y-STRs) or other southern populations (e.g. Vietnamese, Y haplogroups) were also not significantly above zero (Supplementary Tables S6 and S7), as noted previously [13]. In the MDS plots, the Korean samples lay at the border between the northern and southern clusters, rather than within the northern cluster (Figure 2C, D). In order to investigate Y-chromosomal relationships in more detail, we visualized STR haplotypes within a common predominantly northern haplogroup (C*) and southern haplogroup (O3) using networks [50] constructed with the seven Y-STRs common to all datasets (Figure 3). These networks did not show striking geographical structure, so we calculated, for each Korean haplotype, the distance to the closest northern and southern haplotype. In both haplogroups, the mean distance to the southern haplotypes was lower than to the northern haplotypes (C* Korean-north 5.0 steps, Korean-south 4.5 steps; O3 Korean-north 3.5 steps, Korean-south 2.2 steps). This finding is particularly striking for haplogroup C* because it is far more prevalent in the north (Figure 3A).

The genetic differences between the Koreans and other East Asians were examined by AMOVA (Table 4). When samples were grouped into northeast Asians and southeast Asians (excluding Koreans), a highly significant difference was found between the two groups with all markers. Thus there is significant genetic differentiation within the region, and we could then compare each group separately with the Koreans. With mtDNA, Koreans were not significantly different from either group when HVRI sequences were compared, although they were distinct from the southeast Asians in the haplogroup comparisons. With the Y chromosomes, they were again not distinct from either group when haplogroup comparisons were made, but were distinct from the southeast Asians in the STR-based comparison (Table 4).

**Table 4:** AMOVA Results.

Markers	Grouping	Percentage of Variance (*p*-value)		
		Among groups	Among population within groups	Within populations
mtDNA haplogroups	Korean vs. NEAs	−0.03 (0.20332)	1.32 (<0.00001)	98.71 (<0.00001)
	Korean vs. SEAs	2.29 (<0.00001)	2.47 (<0.00001)	95.23 (<0.00001)
	Korean vs. NEAs vs. SEAs	2.16 (<0.00001)	2.51 (<0.00001)	95.33 (<0.00001)
	NEAs vs. SEAs	3.22 (<0.00001)	2.98 (<0.00001)	93.81 (<0.00001)
mtDNA HVRI sequences	Korean vs. NEAs	−0.39 (0.98436)	1.29 (<0.00001)	99.11 (<0.00001)
	Korean vs. SEAs	−0.23 (0.66373)	1.72 (<0.00001)	98.51 (<0.00001)
	Korean vs. NEAs vs. SEAs	2.18 (<0.00001)	2.04 (<0.00001)	95.79 (<0.00001)
	NEAs vs. SEAs	0.26 (<0.00001)	1.58 (<0.00001)	98.16 (<0.00001)
Y-chromosome haplogroups	Korean vs. NEAs	−0.21 (0.46237)	9.43 (<0.00001)	90.78 (<0.00001)
	Korean vs. SEAs	1.34 (0.17889)	10.78 (<0.00001)	87.89 (<0.00001)
	Korean vs. NEAs vs. SEAs	2.89 (<0.00001)	10.35 (<0.00001)	86.77 (<0.00001)
	NEAs vs. SEAs	4.60 (<0.00001)	10.95 (<0.00001)	84.45 (<0.00001)
Y-chromosome STRs	Korean vs. NEAs	3.36 (0.08016)	6.25 (<0.00001)	90.39 (<0.00001)
	Korean vs. SEAs	7.58 (0.00293)	2.48 (<0.00001)	89.94 (<0.00001)
	Korean vs. NEAs vs. SEAs	4.99 (0.00098)	5.65 (<0.00001)	89.36 (<0.00001)
	NEAs vs. SEAs	5.40 (0.00098)	6.82 (<0.00001)	87.78 (<0.00001)

*P* values are obtained by 10,000 permutations.

Our study documents the genetic relationships of the Koreans with their neighboring populations in unprecedented detail. Two major findings emerge. First, the Koreans are overall more similar to northeast Asians than to southeast Asians. This conclusion would be expected from the general correlation between genetic variation and geography observed for human populations, and is supported here by an examination of individual mtDNA haplogroups (Table 2), genetic distances between populations derived from mtDNA or Y-chromosomal data (Figure 2), and the apportionment of genetic diversity between different groups of populations (Table 4). Second, the conclusions from mtDNA and Y-chromosomal analyses differ. Sex-biased admixture is common in human expansions such as that of Bantu-speaking farmers in Africa [58], the spread of the Han ethnic group in China [59] or the post-Columbian peopling of the Americas [60]. The effects in Korea are more subtle, but show a larger male than female contribution from southern East Asia to the population of Korea, most clearly revealed by the admixture estimates, where a 35% contribution from the south was estimated for mtDNA, compared with a 83% contribution for the Y chromosome (Table 5).

**Table 5:** Admixture estimates of Northeast Asians and Southeast Asians in Korean populations.

Markers	Parental contributions	
	Northeast Asians (SD^a^)	Southeast Asians (SD^a^)
MtDNA haplogroups	0.65 (0.25)	0.35 (0.25)
Y-chromosome haplogroups	0.17 (0.14)	0.83 (0.14)
Mt-HG & Y-HG	0.48 (0.21)	0.52 (0.21)

a Standard Deviation.

The predominant genetic relationship with northern East Asians is consistent with other lines of evidence. Xue et al. [31] reported that the northern East Asian populations started to expand in number before the last glacial maximum at 21-18 KYA, while the southern populations all started to expand after it, but then grew faster, and they suggested that the northern populations expanded earlier because they could exploit the abundant megafauna of the “Mammoth Steppe,” while the southern populations could increase in number only when a warmer and more stable climate led to more plentiful plant resources such as tubers. By this criterion, the Koreans, expanding at about 30 KYA [31] also resemble other northern populations. Historical evidence suggests that the Ancient Chosun, the first state-level society, was established in the region of southern Manchuria and later moved into the Pyongyang area of the northwestern Korean Peninsula. Based on archeological and anthropological data, the early Korean population possibly had an origin in the northern regions of the Altai-Sayan and Baikal regions of Southeast Siberia [7], [8], [61].

What could be the origin of the male-biased southern contribution to Korean gene pool illustrated, for example, by haplogroups O-M122 (42.2%) and O-SRY465 (20.1%) [29]. Recent molecular genetic analyses and the geographical distribution of haplogroup O-M122 lineages, found widely throughout East Asia at high frequencies (especially in southern populations and China), have suggested a link between these Y-chromosome expansions and the spread of rice agriculture in East Asia [62]–[64]. In general, Y-chromosomes might be spread via a process of demic diffusion during the early agricultural expansion period [65], [66]. If this interpretation were substantiated, the spatial pattern of Y-haplogroup O would imply a genetic contribution to Korea through the spread of male-mediated agriculture. Large-scale genetic analyses thus begin to reveal some of the complexities of the peopling of Korea, and further studies of individual autosomal loci or genomewide genotyping and sequencing are expected to provide further insights.

## Supporting Information

Table S1 Asian populations studied
(0.05 MB XLS)

Table S2 mtDNA-haplogroup distributions in East Asian populations
(0.05 MB XLS)

Table S3 Y-haplogroup distribution in East Asian populations
(0.03 MB XLS)

Table S4 *F*_ST_ distances of mtDNA haplogroups in east Asian populations (non-significant values are underlined). ^a^Present work; ^b^Kivisild et al. [20]; ^c^Yao et al. [19]; ^d^Lee et al. [30]; ^e^Wen et al. [27]; ^f^Kong et al. [25]; ^g^Li et al. [36].
(0.22 MB XLS)

Table S5 *F*_ST_ values of mtDNA HVR-I Sequences in east Asian populations (non-significant values are underlined). ^a^Present work; ^b^Maruyama et al. [26]; ^c^Kivisild et al. [20]; ^d^Kong et al. [25]; ^e^Yao et al. [19]; ^f^Zhang et al. [28]; ^g^Li et al. [36]; ^h^Powell et al. [37]
(0.07 MB XLS)

Table S6 *F*_ST_ distances of Y chromosome haplogroups in east Asian popoulations (non-significant values are underlined). ^a^Hong et al. [29]; ^b^Xue et al. [31].
(0.05 MB XLS)

Table S7 *R*_ST_ distances in east Asian populations using ten Y chromsome STRs (non-significant values are underlined). ^a^Hara et al. [34]; ^b^Huang et al. [40]; ^c^Hwang et al. [35]; ^d^Yan et al. [38]; ^e^Zhang et al. [33]; ^f^Zhang et al. [39]; ^g^Yong et al. [32]; ^h^Xue et al. [31].
(0.05 MB XLS)
